# Ecological genomics of Chinese wheat improvement: implications in breeding for adaptation

**DOI:** 10.1186/s12870-020-02704-w

**Published:** 2020-10-27

**Authors:** Jie Guo, Chang Li, Junjie Zhao, Jiahui Guo, Weiping Shi, Shunhe Cheng, Meixue Zhou, Chenyang Hao

**Affiliations:** 1 College of Agronomy, Shanxi Agricultural University, Jinzhong, 030801 Shanxi China; 2 Key Laboratory of Crop Gene Resources and Germplasm Enhancement, Ministry of Agriculture and Rural Affairs/The National Key Facility for Crop Gene Resources and Genetic Improvement/Institute of Crop Sciences, Chinese Academy of Agricultural Sciences, Beijing, 100081 China; 3 Key Laboratory of Wheat Biology and Genetic Improvement for Middle and Lower Yangtze Valley, Ministry of Agriculture and Rural Affairs, Lixiahe Agricultural Institute of Jiangsu Province, Yangzhou, 225007 Jiangsu China; 4 Tasmanian Institute of Agriculture, University of Tasmania, Private Bag 1375, Prospect, TAS 7250 Australia

**Keywords:** Bread wheat, Agro-ecological zones, Selection, KASP marker

## Abstract

**Background:**

China has diverse wheat varieties that adapt to very different environments divided into ten agro-ecological zones. A better understanding of genomic differences and patterns of selection among agro-ecological zones could provide useful information in selection of specific adaptive traits in breeding.

**Results:**

We genotyped 438 wheat accessions from ten zones with kompetitive allele specific PCR (KASP) markers specific to 47 cloned genes for grain yield, quality, adaptation and stress resistance. Phylogenetic trees and principle component analysis revealed clear differences in winter and spring growth habits. Nucleotide diversity (*π*) and *π* ratio (*π*_CL_/*π*_MCC_) suggested that genetic diversity had increased during breeding, and that Chinese landraces (CL) from Zones I-V contributed little to modern Chinese cultivars (MCC). *π* ratio and *F*st identified 24 KASP markers with 53 strong selection signals specific to Zones I (9 signals), II (12), III (5), IV (5), V (6), and VI (6). Genes with clear genetic differentiation and strong response to selection in at least three zones were leaf rust resistance gene *Lr34* (I, II, III and IV), photoperiod sensitivity gene *Ppd-D1* (I, II, III, IV and V), vernalization gene *Vrn-B1* (V, VII, VIII and X), quality-related gene *Glu-B1* (I, II and III) and yield-related genes *Sus1-7B* (I, II, III, IV and IX), *Sus2-2A* (I, II, III., IV and VI) and *GW2-6B* (II, V and VI).

**Conclusions:**

This study examined selection of multiple genes in each zone, traced the distribution of important genetic variations and provided useful information for ecological genomics and enlightening future breeding goals for different agro-ecological zones.

**Supplementary information:**

**Supplementary information** accompanies this paper at 10.1186/s12870-020-02704-w.

## Background

China is the largest wheat producer and consumer in the world. The wheat-growing areas are somewhat arbitrarily divided into ten agro-ecological zones each having varieties with different reactions to temperature, photoperiod, and biotic and abiotic stresses [1]. Autumn-sown varieties account for approximately 90% of the production and area across Zones I (4% of the total production area), II (60%), III (13%), IV (10%) and V (minor area of production), whereas spring-sown wheats cover only 7% of the total area across Zones VI, VII and VIII. Zones IX and X have both autumn-sown and spring-sown wheats, but spring-sown wheat in these areas represents only 3% of the total wheat growing area in the country [2].

Currently, a number of genes have been identified and cloned in wheat by positional or map-based cloning, such as *Rht-1* [3], *Vrn-1* [4], *Lr21* [5], *Lr34* [6], *Pm21* [7], and *Fhb1* [8–10]. With advances in sequencing and bioinformatic technologies, comparative genetics led to isolation of several genes regulating grain quality and grain size. The quality-related genes mainly included genes or gene sets for polyphenol oxidase (*PPO*) [11], phytoene synthase (*Psy1*) [12], zeta-carotene desaturase (*Zds1*) [13] and genes encoding high- and low-molecular-weight glutenin subunits [14]. Genes for grain size and grain weight included *TaSus2-2B* [15], *TaCwi-A1* [16], *TaCKX-D1* [17], *TaGW2-6A, 6B* [18–20], *TaSus1* and *TaSus2* [15, 21], *TaGASR-A1* [22], *TaGS-D1* [23, 24], and *TaTGW6* [25]. The most significant practical outcome from cloning of these genes has been the derivation of functional markers that allow identification of those genes/alleles in non-genotyped germplasm or in genetic marker assisted breeding.

Functional markers derived from functional gene motifs and were completely linked to favorable alleles conferring targeted traits [26]. Most importantly, functional markers have the advantage over random DNA markers in that they are not population-specific. To date, more than 150 functional markers in wheat have been developed for over 100 cloned genes for adaptation, grain yield, disease resistance, end-use quality, and tolerance to biotic and abiotic stresses. Many of these markers were subsequently converted into high-throughput KASP assays and were widely adopted in breeding programs [27–29]. Many markers have also been utilized to reveal functions and interactions of alleles at loci such as *Vrn-A1*, *Rht-D1* and *Ppd-B1* [30], to explore natural variation at *Wbm* (bread-making quality), *Glu-B1* (targeting the *Bx7*^*OE*^ allele in particular) and *Sec1* (1B.1R translocation) in global wheat collections [31], and to better understand genetic components of grain yield [32]. A study of 1152 wheat accessions from Asia, Europe, North America and the International Wheat and Maize Center (CIMMYT) that were genotyped using KASP markers designed from 47 genes controlling grain yield, quality, adaptation, and stress tolerance revealed human selection on favorable alleles of multiple genes [33]. These publications collectively demonstrated that KASP markers will be immensely useful for genomics and breeding in wheat.

The past 70 years of wheat breeding in China has witnessed tremendous progress in improvement of grain yield, quality, stress resistance and adaptation. As different agro-ecological zones cover a wide range of latitude and prevailing climatic conditions ranging from high rainfall to desert environments, many different combinations of traits are required for climatic, agronomic and dietary adaptation. A sound understanding of the genetic variation and distribution of most alleles underlying that variation in different zones could provide valuable information for breeding programs not only in China, but also worldwide. In this study, 438 wheat accessions collected from all wheat zones in China were genotyped with 52 functional KASP markers related to grain yield, quality, stress response, and adaptation. Comparisons of the genetic variation between zones revealed patterns of gene flow of key alleles and provided useful information in regard to ecological genomics and suggested future breeding goals for different zones.

## Results

### Population structure of Chinese wheat accessions from the ten agro-ecological zones

**Fig. 1:**
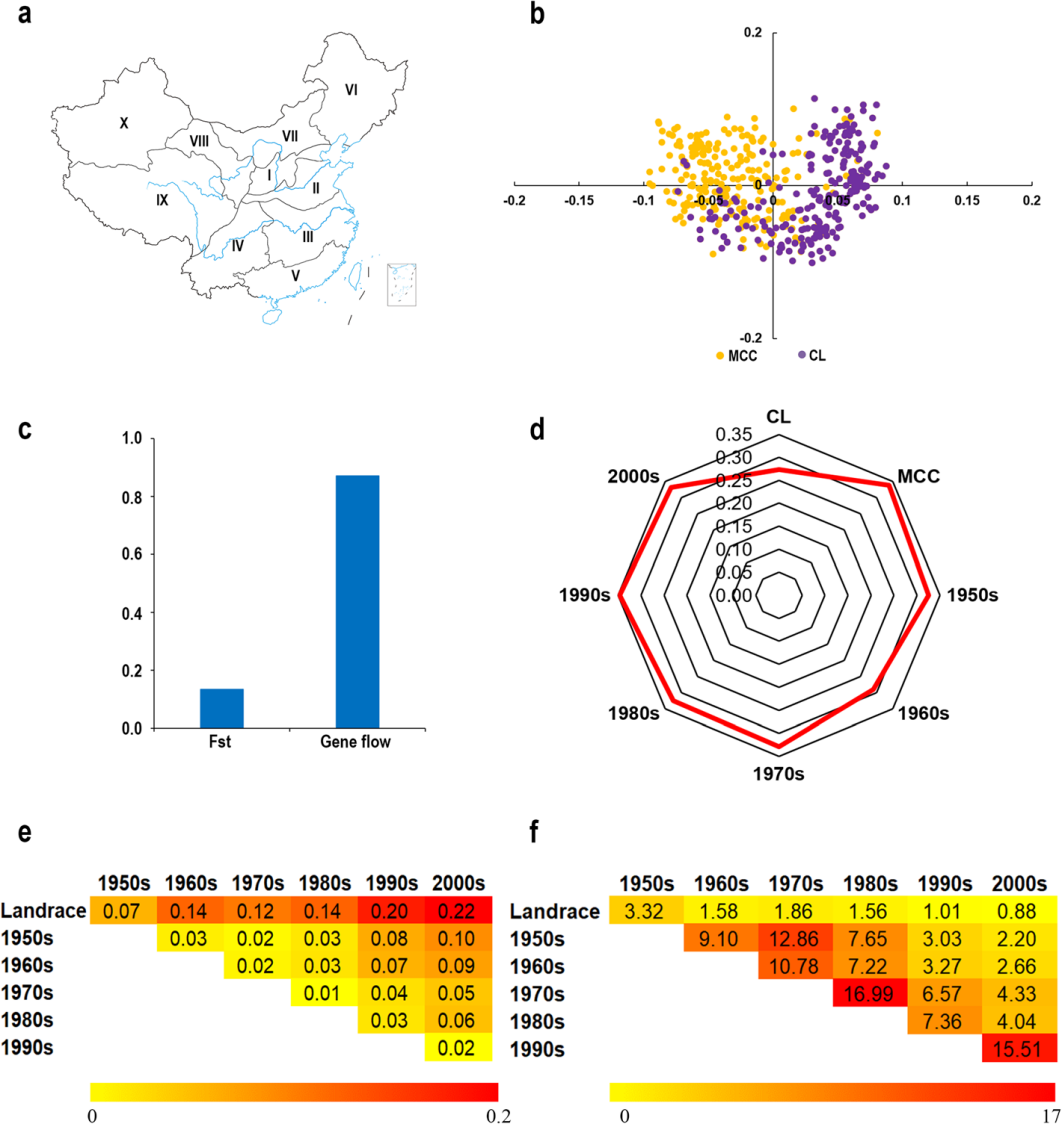
Genetic analysis of 438 wheat accessions with 52 KASP markers. **a**, Ten agro-ecological zones of wheat-growing areas in China. The map information is from the National Geomatics Center of China (http://www.ngcc.cn/ngcc/). **b**, PCA plot of all accessions based on the KASP markers. Chinese landraces (CL) and modern Chinese cultivars (MCC) are shown in purple and orange, respectively. **c**, Genetic differentiation index (*F*st) and gene flow analysis between CL and MCC. **d**, Radar map of genetic diversity (*π* value) of CL and MCC released in different decades. **e**, Heat map of *F*st between CL and MCC released in different decades. The deeper the color, the stronger the differentiation. **f**, Heat map of gene flow between CL and MCC released in different decades. The deeper the color, the stronger the gene flow

Principle component analysis (PCA) divided the accessions into two major subpopulations, namely, Chinese landraces (CL) and modern Chinese cultivars (MCC) (Fig. 1a, b). The mean values of *F*st and gene flow between CL and MCC were 0.13 and 0.87, respectively (Fig. 1c). Nucleotide diversity (*π*) showed that MCC were more diverse than CL (Fig. 1d). Further, compared to the CL, MCC during different decades had higher levels of genetic diversity but lower gene flow (Fig. 1e, f). Among those comparisons, the highest genetic divergence (0.22) and the lowest gene introgression (0.88) occurred in comparison of MCC in the 2000s with CL. Comparing CL and MCC in different decades, *F*st gradually increased while gene flow decreased. Moreover, more introgressions were observed (≥ 10.78) in the 1970s vs 1950s and 1960s, 1970s vs 1980s, and 1990s vs 2000s with the smallest *F*st (≤ 0.02) (Fig. 1e, f), indicating that MCC in adjacent periods had the least *F*st and the largest gene flow, and MCC from one period provided the genetic basis for the following decade.

**Fig. 2:**
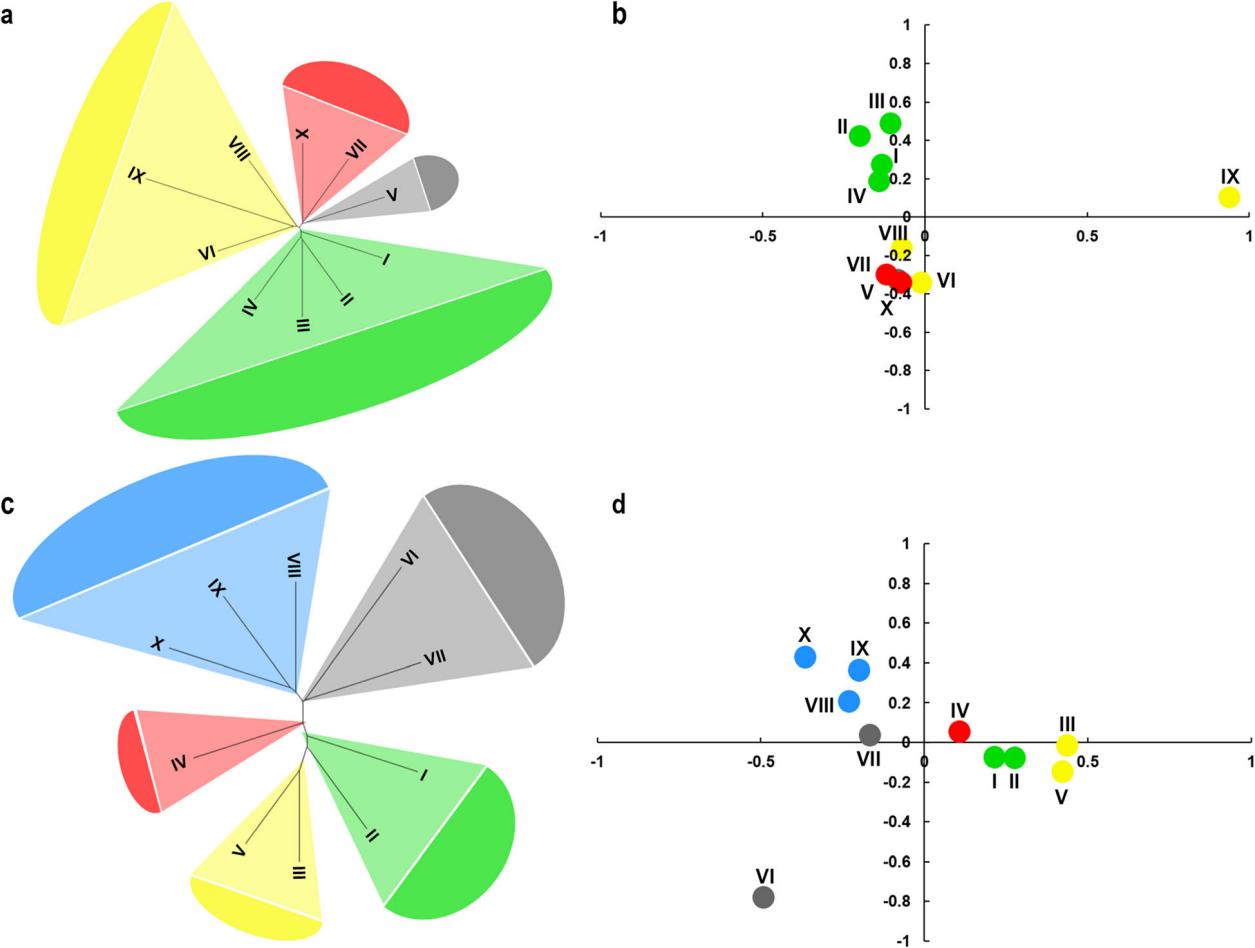
Population structure analysis of ten wheat agro-ecological zones with 52 KASP markers. **a**, Phylogenetic tree of ten wheat agro-ecological zones in Chinese landraces (CL), I to X represents each zone. **b**, PCA plot of ten wheat agro-ecological zones in CL, the solid dot means each zone, and the color of dots are the same with that in Fig. 2a. **c**, Phylogenetic tree of ten wheat agro-ecological zones in modern Chinese cultivars (MCC), I to XI represents each zone. **d**, PCA plot of ten wheat agro-ecological zones in MCC, the solid dot means each zone, and the color of dots are the same with that in Fig. 2c

Population structure analysis of wheat accessions from all zones was further carried out for each subpopulation (Fig. 2). Grouping of CL accessions mainly corresponded to the autumn-sown and spring-sown wheat zones, with Zones I, II, III, IV and V being autumn-sown, and Zones VI, VII VIII, IX and X spring-sown (Fig. 2a, b). Additionally, Zone IX clustered with Zones VI and VIII with relatively large genetic differences among them, but less than those with all other zones. This classification was not as obvious among MCC, but still revealed the separation of autumn and spring-sown wheat (Fig. 2c, d). In contrast to CL, Zones I, II, III, and IV were divided into two subgroups, with Zones I and II clustering together in MCC.

### Genetic diversity and introgression across zones

**Fig. 3:**
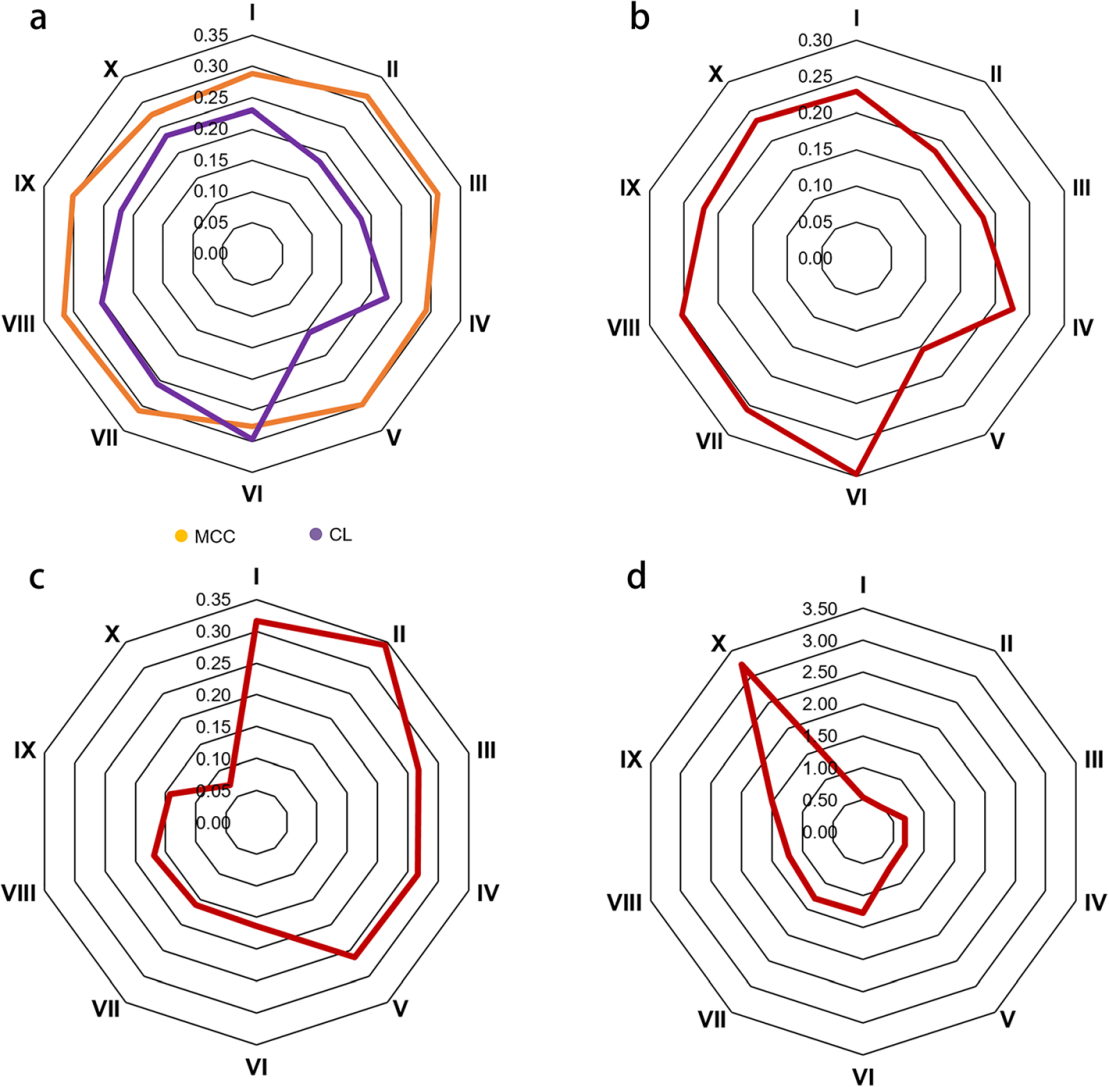
Radar maps of genetic diversity, differentiation and gene flow in ten wheat agro-ecological zones. **a**, Nucleotide diversity (*π* value) of Chinese landraces (CL) and modern Chinese cultivars (MCC) in ten wheat agro-ecological zones. CL and MCC are shown in purple and orange, respectively. **b**, *π* ratio (*π*_CL_/*π*_MCC_) of ten wheat agro-ecological zones. **c**, *F*st between CL and MCC in each wheat agro-ecological zone. **d**, Gene flow between CL and MCC in each zone

Nucleotide diversity analysis (*π* and *π*_CL_/*π*_MCC_) showed that MCC had higher nucleotide diversity than landraces in all zones expect Zone VI. The *π*_CL_ to *π*_MCC_ ratio of < 1 indicated increased nucleotide diversity due to breeding and selection in each zone (Fig. 3a, b; Additional file 1). Comparison between CL and MCC showed that genetic divergence (*F*st = 0.07) was smallest in Zone X but that zone had the highest introgression (3.23). Higher genetic divergence (*F*st ≤ 0.70) and lower genetic introgression (gene flow > 0.26) were observed between CL and MCC in Zones I-V; Zone II had the highest divergence (*F*st = 0.34) but lowest introgression (gene flow = 0.54) (Fig. 3c, d; Additional file 1). The phylogenetic tree also suggested that CL made little contribution to MCC in Zones I-V, whereas it made a significant contribution to wheat breeding and selection in Zone X (Additional file 2).

**Fig. 4:**
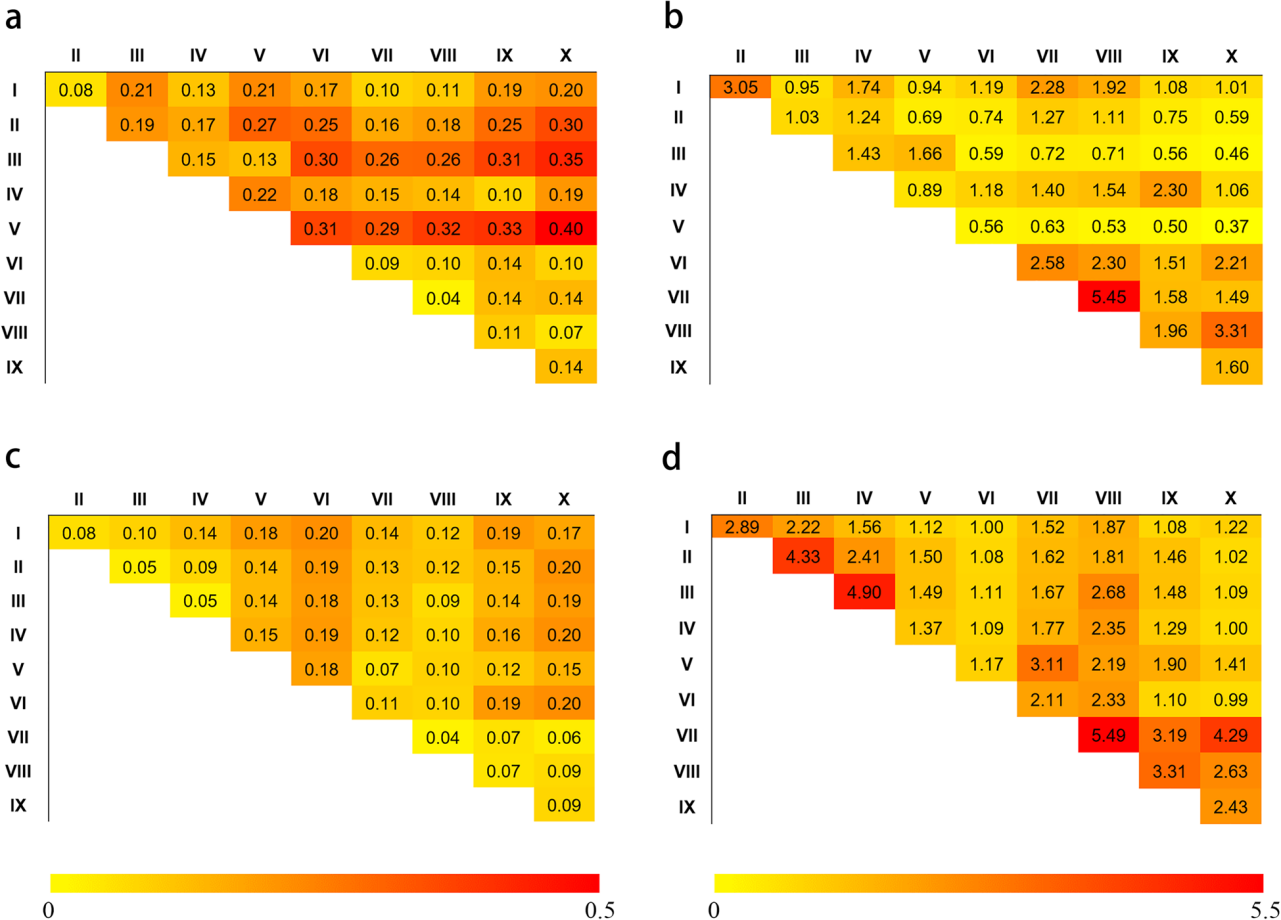
Genetic differentiation and gene flow analysis among ten wheat agro-ecological zones. **a**, Heat map of *F*st among ten wheat agro-ecological zones in Chinese landraces (CL). **b**, Heat map of gene flow among ten wheat agro-ecological zones in CL. **c**, Heat map of *F*st among ten wheat agro-ecological zones in modern Chinese cultivars (MCC). **d**, Heat map of gene flow among ten wheat agro-ecological zones in MCC

Comparing zones to each other, CL had greater genetic divergence and less gene flow than the MCC (Fig. 4). Within CL there were more frequent introgressions (5.45) between Zones VII and VIII with the smallest *F*st (0.04), followed by Zones I and II (3.05 with *F*st = 0.08), and VIII and X (3.31 with *F*st = 0.07). Fewer gene flow events and larger *F*st were observed for comparisons II vs X, III vs VI, IX vs X, and V vs VI, VII, VIII, IX and X, reflecting greater genetic divergence (gene flow < 0.60 and *F*st ≥ 0.30). Among those comparisons, the largest genetic divergence and the least gene introgressions were for Zones V to X. Genetic introgression in CL often occurring between adjacent zones was likely a consequence of their arbitrary classification. For MCC there was less genetic divergence and more frequent introgression among the ten zones (*F*st ≤ 0.20). II vs III, III vs IV, and VII vs VIII and X showed more frequent introgressions (> 4.29) and smaller *F*st (≤ 0.06). More frequent introgressions across zones in MCC suggested that modern breeding had broken through the separation to some extent among agro-ecological zones.

### Selection signals of key genes in all zones

**Fig. 5:**
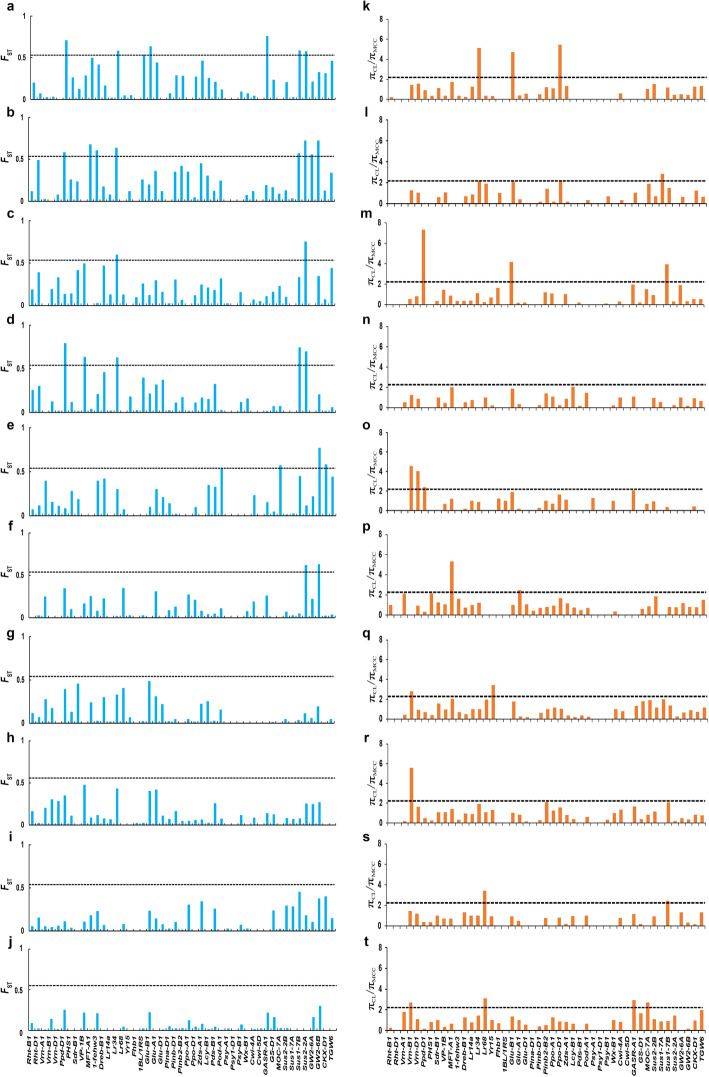
Selective sweeps detected by *F*st and *π* ratio between Chinese landraces (CL) and modern Chinese cultivars (MCC) in ten wheat agro-ecological zones. **a-j**, Selection signals of wheat improvement using 52 KASP markers detected by *F*st between CL and MCC in each zone. **k-t**, Selection signals of wheat improvement using 52 KASP markers detected by *π* ratio (*π*_CL_/*π*_MCC_) in Zones I to X. Horizontal dashed lines indicate significance thresholds of selection signals (top 5%)

Genetic differentiation (*F*st and *π*_CL_/*π*_MCC)_ analysis by comparison of CL with MCC indicating that *Sus2-2A*, *GW2-6B*, *GASR-A1* and *Lr34* had undergone strong selection (significant at α = 0.05) (Additional file 3). Similar analyses for each zone identified 53 loci subjected to strong selection (significant at α = 0.05). In particular, Zones II, I, V, VI, III and IV were under selection with 12, 9, 6, 6, 5 and 5 selective signatures, respectively, compared to Zones VII, VIII, IX and X with 2, 2, 2 and 4 selection signals, respectively. These included some well-known genes strongly selected in more than three zones, including *Lr34* (Zones I, II, III and IV), *Ppd-D1* (I, II, III, IV and V), *Vrn-B1* (V, VII, VIII and X), *Glu-B1* (I, II and III), *Sus1-7B* (I, II, III, IV and IX), *Sus2-2A* (I, II, III, IV and VI) and *GW2-6B* (II, V and VI) (Fig. 5; Additional file 4).

### Allelic distribution of 47 loci across zones

**Fig. 6:**
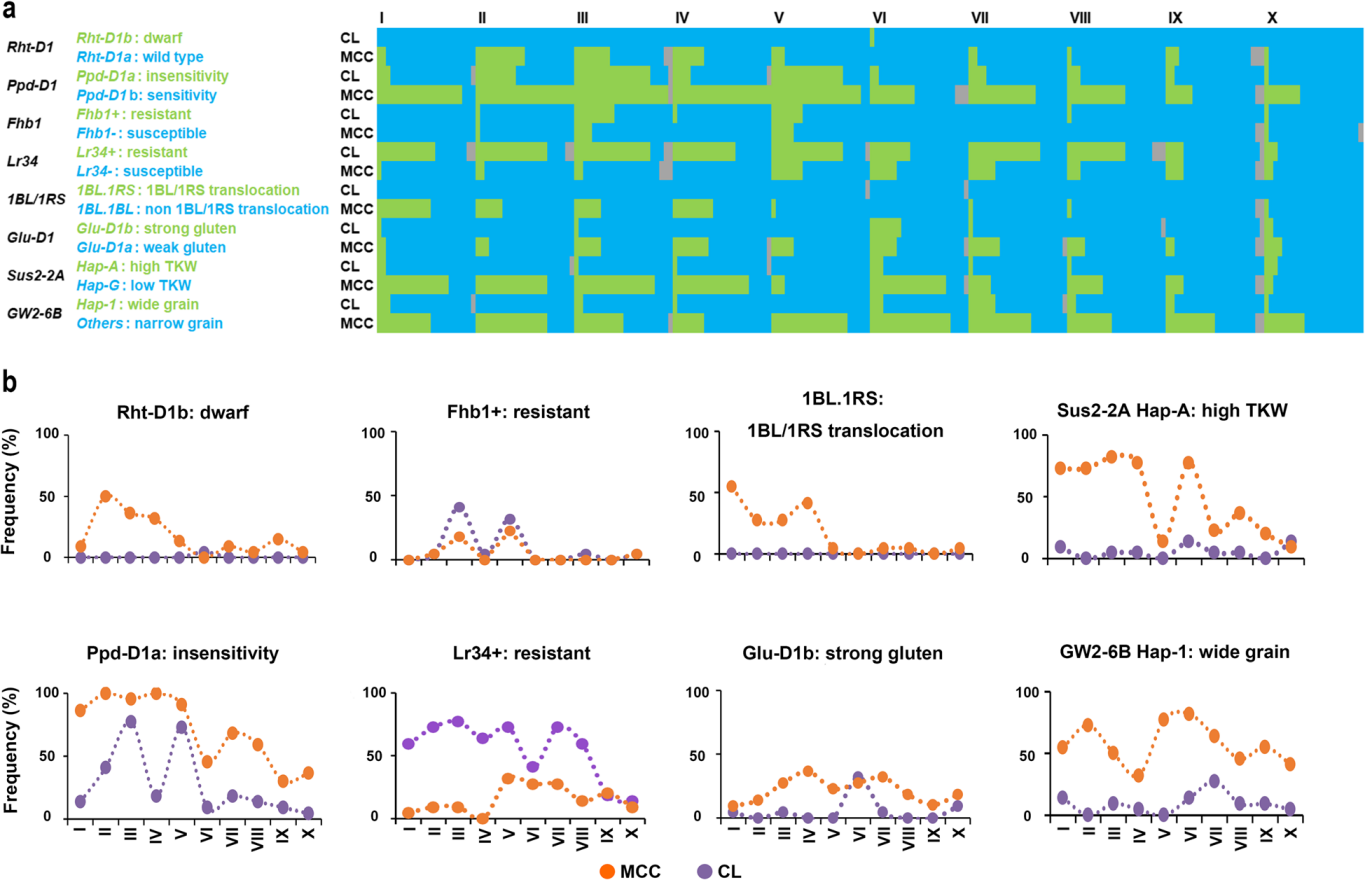
Allele distributions and frequencies of eight genes that underwent selection across the ten wheat agro-ecological zones. **a**, Allele distributions of eight genes in both Chinese landraces (CL) and modern Chinese cultivars (MCC) in each zone. **b**, Allele frequencies of eight genes in both CL and MCC in ten wheat agro-ecological zones. CL and MCC are shown in purple and orange, respectively

Allelic frequencies of most of 47 loci took an uneven distribution in ten agro-ecological wheat zones, obviously in both CL and MCC (Fig. 6; Additional files 5, 6). For adaptation-related genes, the semi-dwarf alleles *Rht-B1b* and *Rht-D1b* in all zones were rarely present in CL, but reached 30% in MCC after the 1990s, with Zone II having the highest frequency (11 accessions, 50%) carrying *Rht-D1b* (Fig. 6b; Additional file 5). About 65% of MCC and CL carried the winter type *vrn-B1* allele in Zones I-IV, whereas 81% had the spring type *Vrn-B1b* allele in Zones VI-X. All accessions from Zone VI carried *Vrn-B1b* (Additional files 3, 5). This distribution of *Vrn* alleles corresponded to the typical growth habit of accessions from each zone (Additional file 7). Among all zones 71% of CL carried the photoperiod sensitive allele *Ppd-D1b*, whereas 71% of MCC carried the contrasting photoperiod insensitive allele *Ppd-D1a*. A very high proportion of MCC (95%) in Zones I-V carried *Ppd-D1a* enabling earlier flowering (and maturity) under lower temperatures in the autumn-sown zones (Fig. 6) where double cropping is a common practice.

For stress resistance genes, 22% of MCC in all zones carried the *MFT-A1* functional marker associated with sprouting resistance allele *PHS*^*+*^, lower than the frequency of 41% for CL with all 22 CL accessions from Zone II carrying the resistance allele; only 14% of MCC in Zone I, 9% in Zone IV, 18% in Zone V, 5% in Zone VI and 14% in Zone VII carried that allele (Additional file 6). Moreover, 55% of the CL carried the slow rusting *Lr34*^+^ allele across all zones, compared with only 15% for MCC (Fig. 6). This explained the relatively high pre-harvest sprouting rates and possibly the wide occurrence of leaf rust in MCC in recent years. About 9% of CL (20 accessions) and 5% of MCC (11 accessions) carried the Fusarium head blight resistance allele *Fhb1*^+^ in all zones, with Zones III and IV having the largest number (25 accessions). Predictably, the 1BL.1RS translocation was not detected in CL across all zones but was present at high frequency (89%) in MCC in Zones I-IV (Fig. 6).

In regard to quality-related genes 45 and 19% of the MCC carried the *Ax1* or *Ax2* alleles at the *Glu-A1* locus and the overexpression allele at *Glu-B1*, respectively, much lower than for CL (83 and 53%). However, more MCC carried the *Glu-D1b* allele (22%) than CL (5%) (Additional files 5, 6). Moreover, 80% of MCC carried the *Pinb-D1b* allele across all zones, compared with 6% for CL. Among MCC carrying *Pinb-D1b* approximately 50% had the allele in Zones I, II and VI (Additional files 5, 6). In the case of *Pds-B1* 22% of the MCC carried allele *b* associated with lower yellow pigment content (YPC) compared to 3% for the CL. Zone IV had the highest frequency (41%) of *Pds-B1b* (Additional files 5, 6)*.* The general trend of MCC carrying larger numbers of quality-related alleles was indicative of strong selection for quality attributes.

For grain morphology 57% of MCC carried haplotype *Hap-1* at the *GW2-6B* locus, much higher than CL (9%). The difference was especially large in Zones II, V and VI where 73, 77 and 82% of the MCC possessed this allele compared to no CL in Zones II and V and 13% in Zone VI (Fig. 6). This indicated that modern wheat breeding selected for larger (wider) grain. For grain weight, an average 76% of MCC in Zones I, II, III, IV and VI carried the *Hap-A* at the *Sus2-2A* locus, much higher than those in CL with an average 5%. Other zones had relatively lower percentages of accessions carrying this allele (5% for CL and 20% for MCC), implying that the major wheat-growing zones select for high grain weight (Fig. 6).

The number of fixed allelic variants (allele frequency ≥ 95%) across all zones except Zone VI showed that the CL had more fixed variations than the MCC (Additional file 8a; Additional file 9). Further, a comparison of numbers of rare alleles (allele frequency < 5%) across all zones revealed more rare alleles in CL than MCC in Zones I, IV, VI, VII, VIII and IX, in contract to Zones V and X where MCC had a higher number of rare alleles, whereas frequencies were similar in Zones II and III, indicating a quantitative difference of rare alleles between CL and MCC across the ten agro-ecological zones (Additional file 8b; Additional file 9).

## Discussion

### Different photoperiod and vernalization alleles for different zones

Selection for high yield and increased adaptation to new environments and multi-cropping systems is a common practice in all wheat breeding programs. Breeding locally adapted cultivars for different agro-ecological zones was more important in the past and required selection of specific adaptation alleles [34]. For example, the photoperiod insensitive allele *Ppd-D1a* was selected in MCC zones with autumn-sowing and a shorter photoperiod. However, this allele was not as frequent in genotypes grown in the spring-sown zones. Another example is the vernalization (*Vrn*) genes. Winter temperatures and length of growing season largely determine the distribution of vernalization alleles. As average January temperatures gradually increased with progression from Zones I to II, II to III, and III to IV, and the length of the growth period decreased [1], and the frequency of *Vrn-B1* and *Vrn-D1* alleles conferring spring type increased (Additional file 6; Additional file 9). The *vrn-A1* allele predominated in the autumn-sown zones, *vrn-D1* was often found in spring wheat accessions in Zones II, III and IV, and *vrn-B1* was frequent in the spring-sown Zones VI, VII and VIII (Additional file 9). As reported by Fu et al. [35] the distributions of day-length and vernalization alleles were largely determined by the severity of the winter temperatures and length of the growing season.

### Introgression of favorable alleles increases genetic diversity of MCC

Applying 52 KASP markers for 47 agriculturally important genes facilitated a better understanding the genetic diversity in landraces and modern cultivars across different wheat zones. Nucleotide diversity was generally higher in MCC than CL in all zones except Zone VI. Gene flow analysis suggested that CL contributed little to the MCC in the major wheat production Zones I-V, consistent with previous studies showing that introduced modern cultivars played a far more important role in wheat production and breeding in China [33]. The higher nucleotide diversity in MCC was attributed to two causes. As the ultimate breeding goal was to increase yield, the frequency of alleles conferring high yield has been rising in modern cultivars as a result of introduced germplasm and breeding [36], which increases genetic diversity. One example was the allele *GW2-6B* (*Hap-1*) for which the average nucleotide diversity was 0.16 in CL and 0.45 in MCC, and the average frequency of *Hap-1* for enhancing grain size increased from 9 to 57% (Figs. 5, 6). The second reason was that hybridization during plant breeding facilitates recombination and exchange of genetic materials, increasing genetic diversity [37]. This increased genetic diversity coincided with the early wheat breeding history in China, when cultivars such as Abbondanza, St 2422/464, Funo and Mentana from Italy were introduced and frequently used in crossing programs. This was followed by the introduction of germplasm with the 1BL/1RS translocation for superior grain yield and disease resistance from Russia and Eastern Europe (Lovrin 10, Predgornaja 2 and Neuzucht) and spring and facultative wheat materials from CIMMYT. In Zones I-IV, 89% of the MCC carried the 1BL/1RS translocation compared to none for the landraces (Fig. 6). Therefore, elite introduced accessions have broadened genetic diversity, advanced cultivar improvement and increased yield and quality attributes in Chinese wheat breeding [1].

### Selection signals provide guidance for future wheat breeding in all agro-ecological zones

The numbers of selection signals were much higher in Zones I-VI than VII-X. This was likely due to the much larger production areas and more intensive breeding efforts in those zones that account for 85% of production, which is most intensive in the Yellow and Huai River valley winter wheat zone (Zone II) with 43% of the wheat area and 60% of production [38]. Selection during domestication and breeding reshapes crop genomes since the effort focused on pyramiding of potentially beneficial alleles located in genic regions [39]. More favorable alleles gradually shifted from minor in terms of frequency to major alleles in both the MCC and CL. For example, alleles for high thousand grain weight, *Sus1-7A-Hap-H* and *Cwi-5D-Hap-C* and alleles for flour color *Psy-A1b, Psy-B1a* or *b* and *Psy-D1a* increased in frequency in both CL and MCC (Additional files 5, 6). These results corroborated previous findings that some alleles for high thousand grain weight and whiteness of flour had become fixed in breeding populations. Frequencies of some other alleles beneficial to production and customer preference, such as *Glu-D1b*, *Pinb-D1b*, *Pds-B1b*, *GW2-6B_Hap-1* and *Sus2-2A*_*Hap-A* have been increasing in modern cultivars due to gradual accumulation from selection in all ten zones (Fig. 6; Additional file 6). However, a few disease resistance alleles such as *Lr68*^+^ and *Yr15*^+^ were relatively rare in both CL and MCC in all zones very likely because the former is not highly effective and seems to be present mainly in South Asian germplasm not widely assessed in China, and *Yr15* is from *Triticum dicoccoides* and not yet widely deployed. Both genes could be future breeding targets.

Cultivar improvement is usually accompanied by both positive and negative effects [36]. In regard to gluten strength, about 91% of CL in Zones I and II carried alleles *Ax1* or Ax2* at the *Glu-A1* locus, far higher than those in MCC with an average 40%. The frequency of *Bx7*^*OE*^ in the MCC were 5 and 14% in Zones I and II, respectively, lower than in CL (68 and 45%) (Additional files 5, 6). Future cultivar improvement could benefit from targeted selection of these alleles. The frequency of pre-harvest sprouting resistance allele of *PHS* at the *MFT-A1* locus was considerably lower in MCC (22%) than CL (41%) across all zones, and was 100% for CL in Zone II. This to some extent explains the reason for the recent high occurrence of pre-harvest sprouting in MCC.

Fusarium head blight or scab has increased in importance in recent years, especially its spread from the more traditional Yangtze River area and southern China to the major production zones in the Yellow and Huai River valley winter wheat zone [40]. Understanding the distribution and putative donors of *Fhb1* in Chinese wheat accessions will facilitate the wider use of this gene and thus contribute to better FHB resistance in China. Here, about 9% of CL (20 accessions) and 5% of MCC (11 accessions) carried *Fhb1*^+^ in all zones, with Zones III and IV having the largest number (25 accessions) (Fig. 6). Chinese wheat breeders commenced research on FHB in the 1950s. Sumai 3 and other cultivars with improved FHB resistance were developed and widely applied in production and breeding programs. Breeding for FHB resistance is a long-term task, but the use of new technologies and resistance sources are likely to lead to improved FHB resistance in new cultivars within the next decade. Further, 55% of the CL carried the slow rusting resistance gene *Lr34* across ten zones, compared with 15% for MCC, frequencies that are consistent with the results of Yang et al. [41] (Fig. 6). To conclude, this analysis of favorable alleles for important agronomic traits in the different wheat zones provides a better understanding of the geographical distribution of key genes nation-wide and will assist molecular breeding.

## Methods

### Plant materials and DNA extraction

The 438 wheat accessions included 22 Chinese landraces (CL) and 22 modern Chinese cultivars (MCC) from each of the ten zones except Zone IX from which 22 CL and 20 MCC were chosen (Fig. 1a; Additional file 7). All accessions were obtained from the Chinese Crop Germplasm Resources Information System (http://www.cgris.net/zhongzhidinggou/index.php). Genomic DNA was extracted from young leaves of each accession using the CTAB method [42].

### KASP genotyping of functional genes

A total of 52 KASP markers for 47 cloned wheat genes described previously were used in this study [27, 33, 43]. These genes were related to yield, quality, disease resistance and adaptation in wheat. Briefly, KASP markers were designed based on the diagnostic SNP markers following standard KASP guidelines. The allele-specific primers used are listed in Additional file 10, and a common reverse primer was designed to ensure that total amplicons were less than 120 bp. KASP assays were performed in 384-well formats with 5.0 μL mixtures containing 2.2 μL of 40 ng/μL DNA, 2.5 μL of 1 × KASP V4.02 × Master mix (KBS-1016-017), 0.04 μL Mg^2+^, 0.056 μL of primer mixture, and 0.204 ddH_2_O. Ultrapure water was used as the non-template control (NTC). PCR cycles of KASP assay were: (1) 94 °C for 15 min; (2) 95 °C for 20s; 65 °C for 25 s initially and the following each cycle decreasing 1 °C for 10 cycles; (3) 95 °C for 10s; 56 °C for 1 min for 30 cycles. QuantStudioTM7 Flex (Applied Biosystems by Life Technologies) was used to collect fluorescence signals for genotyping. Data were visualized and generated with QuantStudioTM Real-time PCR Software v1.3 (Applied Biosystems by Life Technologies).

### Population structure and phylogenetic analysis

Nei’s genetic distances were calculated based on data from the KASP marker assays on all 438 accessions [44]. Neighbor joining (NJ) trees were constructed using PowerMarker v3.25 [45] and visualized using MEGA5 [46]. Principal component analysis (PCA) was applied to all accessions using Adegenet v2.0.1 in R [47].

### Genetic differentiation and gene flow evaluation

Fixation indices (*F*st) and genetic distances were calculated to evaluate population differentiation [48]. Nucleotide diversity values of Tajima’s *π* and gene flow among subpopulations from different zones were analyzed using POPGENE software [49, 50]. To detect improvement-related loci, selection signals were identified by changes in allelic frequencies at polymorphic loci of target genes [51].

## Supplementary information

**Additional file 1: Table S1.** Genetic diversity, *F*st and gene flow in Chinese landraces (CL) and modern Chinese cultivars (MCC) in ten wheat agro-ecological zones.

**Additional file 2: Figure S1.** Phylogenetic tree of wheat accessions in ten wheat agro-ecological zones. a-j, Phylogenetic tree for 438 wheat accessions using 47 KASP markers in Zones I to X. CL and MCC are shown in purple and orange, respectively.

**Additional file 3: Figure S2.** Selective sweeps detected by comparisons between Chinese landraces (CL) and modern Chinese cultivars (MCC). Selection signals of wheat improvement using 47 KASP markers detected by both *F*st (**a**) and *π*_CL_*/π*_MCC_ (**b**) between CL and MCC. Horizontal dashed lines indicate significance thresholds of selection signals (top 5%).

**Additional file 4: Table S2**. Genetic diversity and *F*st for 47 polymorphic genes between Chinese landraces (CL) and modern Chinese cultivars (MCC) in ten wheat agro-ecological zones.

**Additional file 5: Figure S3**. Allele distributions of 39 agronomic genes in Chinese landraces (CL) and modern Chinese cultivars (MCC) in ten wheat agro-ecological zones.

**Additional file 6: Figure S4.** Allele frequencies of 39 agronomic genes in Chinese landraces (CL) and modern Chinese cultivars (MCC) in ten wheat agro-ecological zones. CL and MCC are shown in purple and orange, respectively.

**Additional file 7: Table S3.** Genotypic information regarding the 438 wheat accessions used in this study.

**Additional file 8: Figure S5**. Numbers of allelic variants in Chinese landraces (CL) and modern Chinese cultivars (MCC) in each wheat agro-ecological zone. **a**, Number of fixed variations in CL and MCC in each wheat agro-ecological zone. **b**, Number of rare alleles in CL and MCC in each wheat agro-ecological zone.

**Additional file 9: Table S4.** Frequencies of allelic variations of 47 polymorphic genes in Chinese landraces (CL) and modern Chinese cultivars (MCC) in ten wheat agro-ecological zones.

**Additional file 10: Table S5.** Allelic variations and primer sequences for the 52 KASP assays.

## Data Availability

The dataset supporting the conclusions of this article is included within the Additional files.

## Abbreviations

I: Northern winter wheat region
II: Huai River valley winter wheat region
III: Lower and middle Yangtze River valley winter wheat region
IV: Southwestern winter wheat region
V: Southern winter wheat region
VI: Northeastern spring wheat region
VII: Northern spring wheat region
VIII: Northwestern spring wheat region
IX: Qinghai-Tibet spring-winter wheat region
X: Xinjiang winter-spring wheat region
CL: Chinese landraces
MCC: Modern Chinese cultivars
KASP: Kompetitive allele specific PCR
*π*: Nucleotide diversity
*F*st: Fixation indices
PCA: Principle component analysis
NJ: Neighbor joining

## References

[1] Zhuang QS (2003). Chinese wheat improvement and pedigree analysis.

[2] Zhang XK, Xiao YG, Zhang Y, Xia XC, Dubcovsky J, He ZH (2008). Allelic variation at the vernalization genes *Vrn-A1*, *Vrn-B1*, *Vrn-D1*, and *Vrn-B3* in Chinese wheat cultivars and their association with growth habit. Crop Sci.

[3] Peng J, Richards DE, Hartley NM, Murphy GP, Devos KM, Flintham JE (1999). 'Green revolution' genes encode mutant gibberellin response modulators. Nature..

[4] Yan LL, Loukoianov A, Tranquilli G, Helguera M, Fahima T, Dubcovsky J (2003). Positional cloning of the wheat vernalization gene *VRN1*. Proc Natl Acad Sci U S A.

[5] Huang L, Brooks SA, Li W, Fellers JP, Trick HN, Gill BS (2003). Mapbased cloning of leaf rust resistance gene *Lr21* from the large and polyploid genome of bread wheat. Genetics..

[6] Krattinger SG, Lagudah ES, Spielmeyer W, Singh RP, HuertaEspino J, McFadden H (2009). A putative ABC transporter confers durable resistance to multiple fungal pathogens in wheat. Science..

[7] Cao AZ, Xing LP, Wang XY, Yang XM, Wang W, Sun YL (2011). Serine/threonine kinase gene *Stpk-V*, a key member of powdery mildew resistance gene *Pm21*, confers powdery mildew resistance in wheat. Proc Natl Acad Sci U S A.

[8] Rawat N, Pumphrey MO, Liu SX, Zhang XF, Tiwari VK, Ando K (2016). Wheat *Fhb1* encodes a chimeric lectin with agglutinin domains and a pore-forming toxin-like domain conferring resistance to Fusarium head blight. Nat Genet.

[9] Li GQ, Zhou JY, Jia HY, Gao ZX, Fan M, Luo YJ (2019). Mutation of a histidine-rich calcium-binding-protein gene in wheat confers resistance to Fusarium head blight. Nat Genet.

[10] Su ZQ, Bernardo A, Tian B, Chen H, Wang S, Ma HX (2019). A deletion mutation in *TaHR* confers *Fhb1* resistance to Fusarium head blight in wheat. Nat Genet.

[11] He XY, He ZH, Zhang LP, Sun DJ, Morris CF, Fuerst EP (2007). Allelic variation of polyphenol oxidase (PPO) genes located on chromosomes 2A and 2D and development of functional markers for the PPO genes in common wheat. Theor Appl Genet.

[12] He XY, He ZH, Ma W, Appels R, Xia XC (2009). Allelic variants of phytoene synthase 1 (*Psy1*) genes in Chinese and CIMMYT wheat cultivars and development of functional markers for flour colour. Mol Breed.

[13] Zhang CY, Dong CH, He XY, Zhang LP, Xia XC, He ZH (2011). Allelic variants at the *TaZds-D1* locus on wheat chromosome 2DL and their association with yellow pigment content. Crop Sci.

[14] Jin H, Yan J, Peña RJ, Xia XC, Morgounov A, Han LM (2011). Molecular detection of high- and low-molecular-weight glutenin subunit genes in common wheat cultivars from 20 countries using allele-specific markers. Crop Pasture Sci.

[15] Jiang QY, Hou J, Hao CY, Wang LF, Ge HG, Dong YS (2011). The wheat (*T. aestivum*) sucrose synthase 2 gene (*TaSus2*) active in endosperm development is associated with yield traits. Funct Integr Genomics.

[16] Ma DY, Yan J, He ZH, Wu L, Xia XC (2012). Characterization of a cell wall invertase gene *TaCwi-A1* on common wheat chromosome 2A and development of functional markers. Mol Breed.

[17] Zhang L, Zhao YL, Gao LF, Zhao GY, Zhou RH, Zhang BS (2012). *TaCKX6-D1*, the ortholog of rice *OsCKX2*, is associated with grain weight in hexaploid wheat. New Phytol.

[18] Su ZQ, Hao CY, Wang LF, Dong YC, Zhang XY (2011). Identification and development of a functional marker of *TaGW2* associated with grain weight in bread wheat (*Triticum aestivum* L.). Theor Appl Genet.

[19] Qin L, Hao CY, Hou J, Wang YQ, Li T, Wang LF (2014). Homologous haplotypes, expression, genetic effects and geographic distribution of the wheat yield gene *TaGW2*. BMC Plant Biol.

[20] Qin L, Zhao JJ, Li T, Hou J, Zhang XY, Hao CY (2017). *TaGW2*, a good reflection of wheat polyploidization and evolution. Front Plant Sci.

[21] Hou J, Jiang QY, Hao CY, Wang YQ, Zhang HN, Zhang XY (2014). Global selection on sucrose synthase haplotypes during a century of wheat breeding. Plant Physiol.

[22] Dong LL, Wang FM, Liu T, Dong ZY, Li AL, Jing RL (2014). Natural variation of *TaGASR7-A1* affects grain length in common wheat under multiple cultivation conditions. Mol Breed.

[23] Zhang YJ, Liu JD, Xia XC, He ZH (2014). *TaGS-D1*, an ortholog of rice *OsGS3*, is associated with grain weight and grain length in common wheat. Mol Breed.

[24] Ma L, Li T, Hao CY, Wang YQ, Chen XH, Zhang XY (2016). *TaGS5-3A*, a grain size gene selected during wheat improvement for larger kernel and yield. Plant Biotechnol J.

[25] Hanif M, Gao FM, Liu JD, Wen WE, Zhang YJ, Rasheed A (2016). *TaTGW6-A1*, an ortholog of rice *TGW6*, is associated with grain weight and yield in bread wheat. Mol Breed.

[26] Andersen JR, Lübberstedt T (2003). Functional markers in plants. Trends Plant Sci.

[27] Rasheed A, Wen WE, Gao FM, Zhai SN, Jin H, Liu JD (2016). Development and validation of KASP assays for genes underpinning key economic traits in bread wheat. Theor Appl Genet.

[28] Rasheed A, Xia XC (2019). From markers to genome-based breeding in wheat. Theor Appl Genet.

[29] Rasheed A, Takumi S, Hassan MA, Imtiaz M, Ali M, Morgunov AI (2020). Appraisal of wheat genomics for gene discovery and breeding applications: a special emphasis on advances in Asia. Theor Appl Genet.

[30] Khalid M, Afzal F, Gul A, Amir R, Subhani A, Ahmed Z (2019). Molecular characterization of 87 functional genes in wheat diversity panel and their association with phenotypes under well-watered and water-limited conditions. Front Plant Sci.

[31] Rasheed A, Jin H, Xiao YG, Zhang Y, Hao YF, Zhang Y (2019). Allelic effects and variations for key bread-making quality genes in bread wheat using high-throughput molecular markers. J Cereal Sci.

[32] Sehgal D, Mondal S, Guzman C, Garcia Barrios G, Franco C, Singh R (2019). Validation of candidate gene-based markers and identification of novel loci for thousand-grain weight in spring bread wheat. Front Plant Sci.

[33] Zhao JJ, Wang ZW, Liu HX, Zhao J, Li T, Hou J (2019). Global status of 47 major wheat loci controlling yield, quality, adaptation and stress resistance selected over the last century. BMC Plant Biol.

[34] Yang FP, Zhang XK, Xia XC, Laurie DA, Yang WX, He ZH (2009). Distribution of the photoperiod insensitive *Ppd-D1a* allele in Chinese wheat cultivars. Euphytica..

[35] Fu DL, Szűcs P, Yan LL, Helguera M, Skinner JS, Zitzewitz JV (2005). Large deletions within the first intron in *VRN-1* are associated with spring growth habit in barley and wheat. Mol Gen Genomics.

[36] Gao LF, Zhao GY, Huang DW, Jia JZ (2017). Candidate loci involved in domestication and improvement detected by a published 90K wheat SNP array. Sci Rep.

[37] Hao CY, Wang YQ, Chao S, Li T, Liu HX, Wang LF (2017). The iSelect 9 K SNP analysis revealed polyploidization induced revolutionary changes and intense human selection causing strong haplotype blocks in wheat. Sci Rep.

[38] He ZH, Rajaram S, Xin ZY, Huang GZ (2001). A history of wheat breeding in China.

[39] Shi JP, Lai JS (2015). Patterns of genomic changes with crop domestication and breeding. Curr Opin Plant Biol.

[40] Zhu ZW, Xu DA, Cheng SH, Gao CB, Xia XC, Hao YF (2018). Characterization of Fusarium head blight resistance gene *Fhb1* and its putative ancestor in Chinese wheat germplasm. Acta Agron Sin.

[41] Yang WX, Yang FP, Liang D, He ZH, Shang XW, Xia XC (2008). Molecular characterization of slow-rusting genes *Lr34*/*Yr18* in Chinese wheat cultivars. Acta Agron Sin.

[42] Chen DH, Ronald PC (1999). A rapid DNA mini preparation method suitable for AFLP and other PCR applications. Plant Mol Biol Rep.

[43] Liu Y, He ZH, Appels R, Xia XC (2012). Functional markers in wheat: current status and future prospects. Theor Appl Genet.

[44] Nei M, Tajima F, Tateno Y (1983). Accuracy of estimated phylogenetic trees from molecular data. II gene frequency data. J Mol Evol.

[45] Liu KJ, Muse SV (2005). PowerMarker: an integrated analysis environment for genetic marker analysis. Bioinformatics..

[46] Tamura K, Peterson D, Peterson N, Stecher G, Nei M, Kumar S (2011). MEGA5: molecular evolutionary genetics analysis using maximum likelihood, evolutionary distance, and maximum parsimony methods. Mol Biol Evol.

[47] Jombart T, Devillard S, Balloux F (2010). Discriminant analysis of principal components: a new method for the analysis of genetically structured populations. BMC Genet.

[48] Hudson RR, Slatkin M, Maddison WP (1992). Estimation of levels of gene flow from DNA sequence data. Genetics..

[49] Tajima F (1989). Statistical method for testing the neutral mutation hypothesis by DNA polymorphism. Genetics..

[50] Yeh FC, Yang RC, Boyle TB, Ye ZH, Mao JX, Yeh C (1999). Popgene version 1.32: the user friendly software for population genetic analysis.

[51] Doebley JF, Gaut BS, Smith BD (2006). The molecular genetics of crop domestication. Cell..

