# Supplementary figures and images for: Ecological genomics of Chinese wheat improvement: implications in breeding for adaptation
Source: BMC Plant Biol. 2020 Oct 27;20:494. doi: 10.1186/s12870-020-02704-w (PMC7590805; doi:10.1186/s12870-020-02704-w)

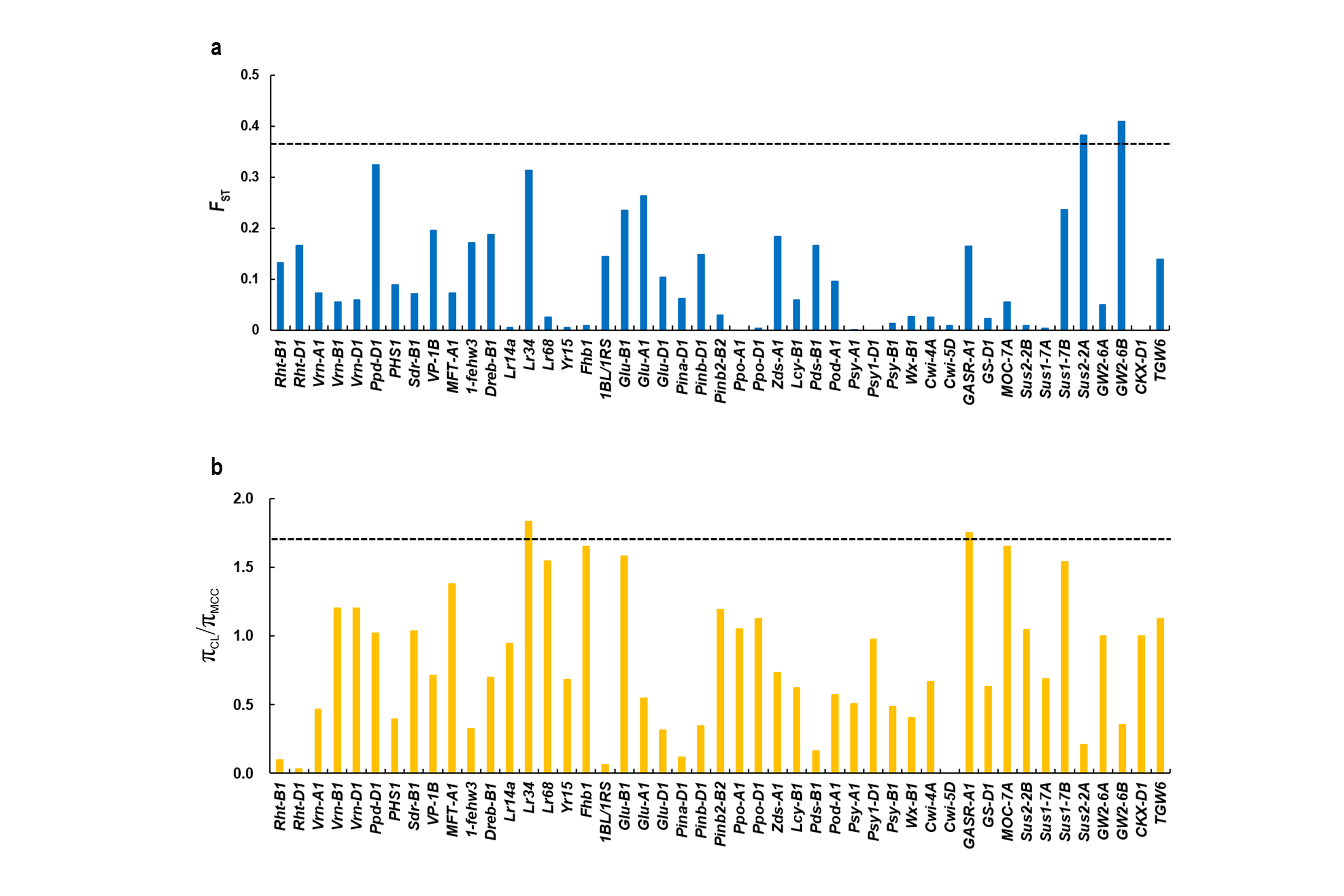

Supplement: Supplementary file 3 — Additional file 3: Figure S2. Selective sweeps detected by comparisons between Chinese landraces (CL) and modern Chinese cultivars (MCC). Selection signals of wheat improvement using 47 KASP markers detected by both Fst (a) and πCL/πMCC (b) between CL and MCC. Horizontal dashed lines indicate significance thresholds of selection signals (top 5%). [file 12870_2020_2704_MOESM3_ESM.tif]

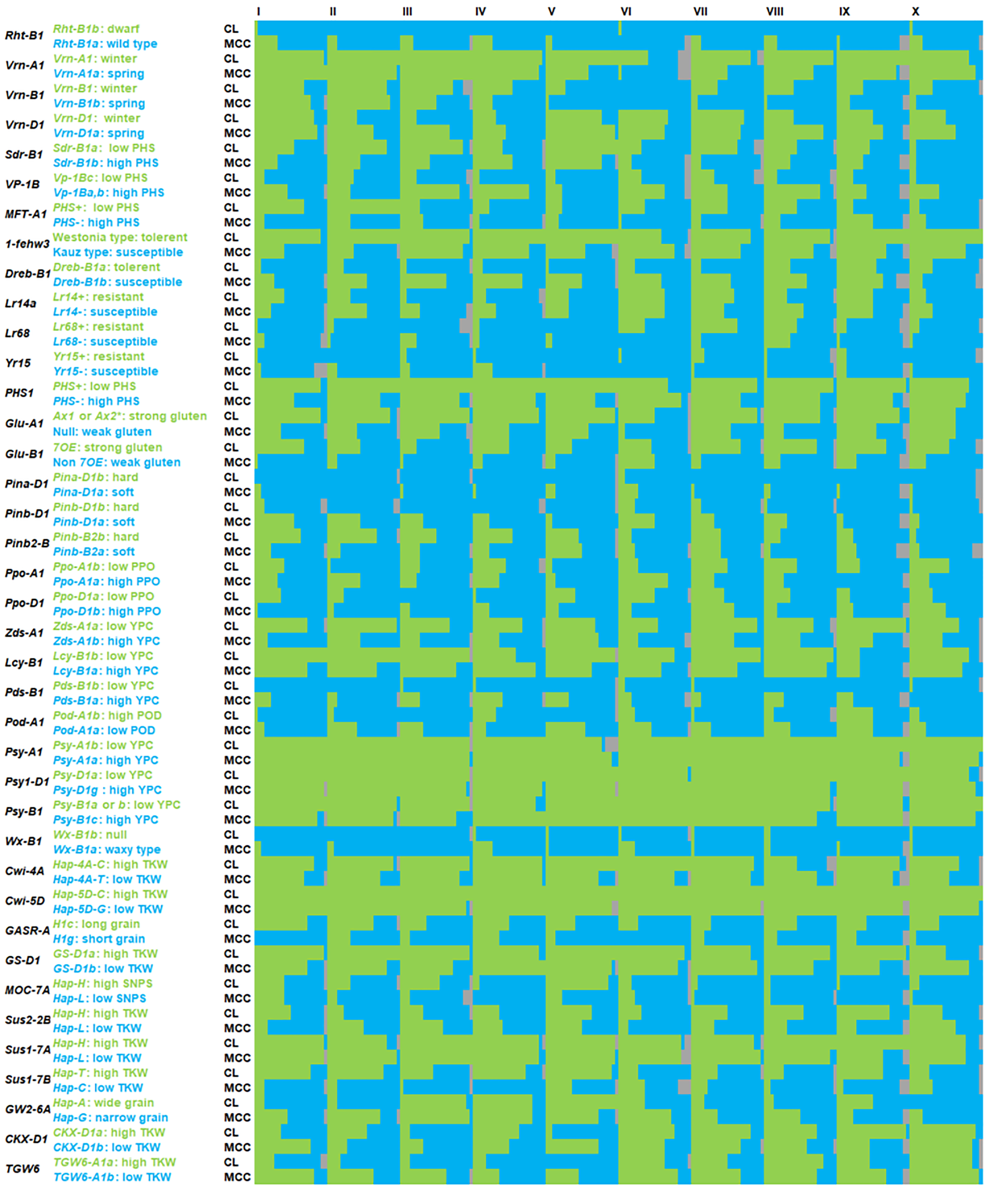

Supplement: Supplementary file 5 — Additional file 5: Figure S3. Allele distributions of 39 agronomic genes in Chinese landraces (CL) and modern Chinese cultivars (MCC) in ten wheat agro-ecological zones. [file 12870_2020_2704_MOESM5_ESM.tif]

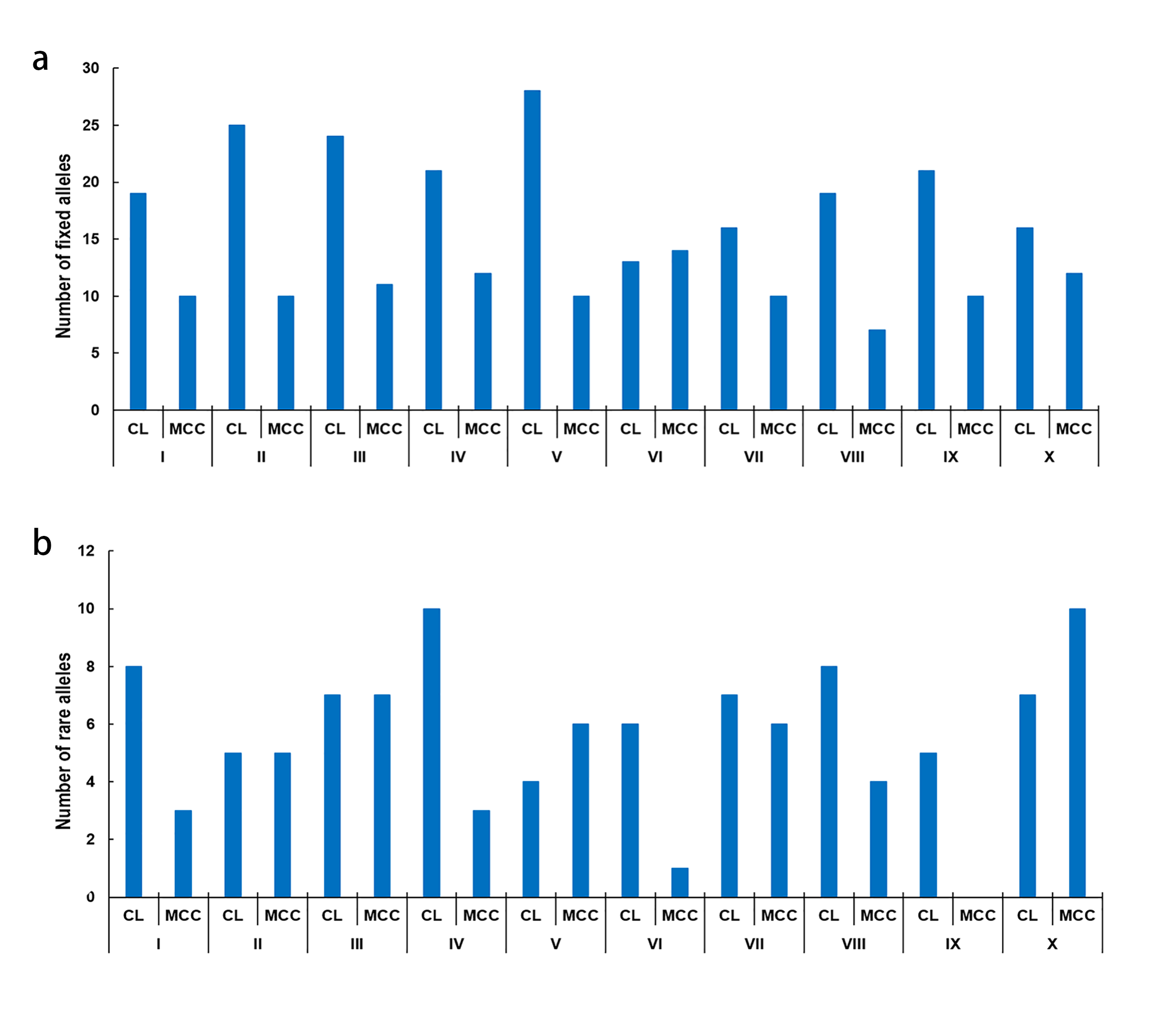

Supplement: Supplementary file 8 — Additional file 8: Figure S5. Numbers of allelic variants in Chinese landraces (CL) and modern Chinese cultivars (MCC) in each wheat agro-ecological zone. a, Number of fixed variations in CL and MCC in each wheat agro-ecological zone. b, Number of rare alleles in CL and MCC in each wheat agro-ecological zone. [file 12870_2020_2704_MOESM8_ESM.tif]
